# An Approach to the Child with Acute Glomerulonephritis

**DOI:** 10.1155/2012/426192

**Published:** 2011-11-24

**Authors:** Thomas R. Welch

**Affiliations:** Department of Pediatrics, Upstate Golisano Children's Hospital, One Children's Circle, SUNY Upstate Medical University, Syracuse, NY 13210, USA

## Abstract

Acute glomerulonephritis (AGN) is a common condition in childhood. Many children with AGN can be managed in the primary care setting. The diagnosis is usually made on the basis of urinary findings, especially the presence of red blood cell casts. One of the most important initial investigations is determining the complement C3 level; hypocomplementemia is most characteristic of post streptococcal AGN, while normocomplementemia is most often seen with IgA nephropathy. Children whose AGN is accompanied by significant hypertension or renal insufficiency should be assessed by a specialist immediately. The presence of serious extrarenal signs or symptoms also merits urgent referral. Otherwise, serial followup in the primary care office is appropriate.

## 1. Introduction

Many children with acute glomerulonephritis (AGN) are first seen in their primary physicians' offices. This initial contact may be crucial in determining the child's most appropriate disposition as well as identifying any immediate threats to life. 

This paper will review the office approach to AGN in children on the basis of upon a firm grounding in pathophysiology. It will begin with an overview of the pathology and pathophysiology of glomerulonephritis and then present a practical outline of the important aspects of the history and physical examination pertinent to a child with suspected AGN. It will then provide guidance in choosing and interpreting appropriate laboratory studies for the initial evaluation. Finally, some guidance will be provided on referral of children with AGN, including a discussion of some situations in which management by the primary caretaker may be appropriate.

## 2. Overview of AGN

AGN is a complex of findings which is marked histologically by a generalized glomerular inflammation. Frequently, renal biopsy is not available, but AGN can usually be recognized by the clinical picture of hematuria, fluid overload (edema and hypertension), and some evidence of renal insufficiency (elevation of BUN and creatinine). 

In most circumstances, glomerular inflammation begins with an antigen-antibody reaction, either direct antibody binding to an antigen expressed or trapped in the glomerulus, or the localization of a circulating complex in the kidney. This incites injury by activating one or more systems of inflammatory mediators: the complement cascade, coagulation factors, cytokines, growth factors, and others. The inflammation is marked by proliferation of resident glomerular cells and infiltration by lymphocytes or neutrophils. 

The glomerular inflammation and expansion impairs the microcirculation, reducing the glomerular filtration rate (GFR) and usually resulting in an increase in BUN and creatinine. This reduction in GFR, in turn, leads to the retention of salt and water, causing fluid overload. The degree of fluid overload in AGN can vary considerably. In severe situations, it can be manifest by life-threatening hypertension and pulmonary edema. Indeed, hypertensive encephalopathy may be the presenting complaint in some children with AGN [1]. 

In some situations, AGN is a primary process, and virtually, all of the clinical findings are a consequence of the renal lesion. Poststreptococcal AGN is the best example of this [2]. In other cases, the AGN is but one manifestation of a systemic illness which has targeted multiple organs, each of which may be independently injured. In children, the AGN associated with Henoch Schoenlein purpura is the prototype for this [3]. 

Fortunately, most cases of AGN in children are either self-limited or amenable to therapy although there may be devastating complications of the illness during the acute phase. Less commonly, what begins as an apparent AGN may presage the development of a chronic process, which ultimately may progress into irreversible end-stage renal disease (ESRD).

## 3. History and Physical Examination

Most typically, the child with AGN will be seen because of the sudden development of change in urine color. On occasion, however, the presenting complaint may relate to a complication of the disease: hypertensive seizures, edema, and so forth.

The history begins with obtaining more details about the change in urine. Hematuria in children with AGN is typically described as “coke,” “tea,” or “smoky” colored. True bright red blood in the urine is more likely a consequence of anatomic problems such as urolithiasis [4] than glomerulonephritis. Urine color in AGN is uniform throughout the stream. The gross hematuria of AGN is virtually always painless; dysuria accompanying gross hematuria points to acute hemorrhagic cystitis [5] rather than renal disease. A history of previous such episodes would point to an exacerbation of a chronic process such as IgA nephropathy [6]. Although a history of a recent documented streptococcal infection would be consistent with poststreptococcal AGN, such a history is frequently unavailable. 

It is next important to ascertain any symptoms suggestive of complications of the AGN. These might include shortness of breath or exercise intolerance from fluid overload or headaches, visual disturbances, or alteration in mental status from hypertension. 

Since AGN may be the presenting complaint of a multisystem illness, a complete review of systems is vital. Particular attention should be paid to rash, joint discomfort, recent weight change, fatigue, appetite changes, respiratory complaints, and recent medication exposure. The family history should address the presence of any family members with autoimmune disorders, as children with both SLE and membranoproliferative glomerulonephritis (MPGN) may have such relatives. A family history of renal failure (specifically asking about dialysis and kidney transplantation) may be the first clue to a process such as Alport syndrome, which may initially present with an AGN picture. 

The physical examination begins with a careful assessment of vital signs, particularly blood pressure. Blood pressures 5 mm above the 99th percentile for the child's age, sex, and height, especially if accompanied by any alteration in mental status, demand prompt attention. Tachycardia and tachypnea point toward symptomatic fluid overload. Careful examination of the nose and throat may provide evidence of bleeding, suggesting the possibility of one of the ANCA-positive vasculitides such as Wegner's granulomatosis [7]. Cervical lymphadenopathy may be the residua of a recent streptococcal pharyngitis. The cardiopulmonary examination will provide evidence of fluid overload or the pulmonary involvement characterizing the rare kidney-lung syndromes. The abdominal examination is particularly important. Ascites may be present if there is a nephrotic component to the AGN. Hepato- or splenomegaly may point to a systemic disorder. Significant abdominal pain may accompany HSP. Scrotal edema may occur in nephrotic syndrome as well, and orchitis is an occasional finding in HSP. 

A very careful examination of the skin is important in AGN. The rash of HSP, while characteristic when florid, may initially be subtle and limited to the buttocks or the dorsa of the feet. Some peripheral edema from salt and water retention is seen in AGN, but this tends to be a more subtle “brawny” edema than the pitting edema characteristic of nephrotic syndrome. 

Joint involvement occurs in some multisystem disorders with AGN. Small joint (e.g., fingers) is more typical of SLE, while or knee involvement is seen with HSP.

## 4. Laboratory Assessment

Obviously, a good urinalysis is the first order of business in assessing a child with suspected AGN. The presence of red blood cell casts, while not invariably seen, is diagnostic of glomerulonephritis if present [8]. AGN is an inflammatory process, so it is not at all unusual to see white blood cells in nephritic urine. Unfortunately, this occasionally leads to an inappropriate diagnosis of urinary tract infection. 

Proteinuria is also nearly invariant in AGN although any cause of gross hematuria can lead to some urinary protein. If the urine is not grossly bloody, however, the combined presence of hematuria and proteinuria virtually always means glomerulonephritis. 

The initial blood work required in suspected AGN is actually limited; more sophisticated immunologic investigations, for example, are really “second tier” studies after the initial results are known. Obviously, assessing renal function and electrolytes is an important first step, as is obtaining a hemogram. A mild degree of anemia is frequently seen with AGN and likely is dilutional; more significant anemia would be evidence that the process may be more chronic. There are typically no important changes in the white blood cell count or platelet count in most causes of AGN. A normal platelet count in the presence of petechiae and purpura is the usual finding in HSP. 

Beyond these basic tests, only a few others are helpful in the initial evaluation. A serum albumin is usually included; a slight degree of hypoalbuminemia is typical of many inflammatory processes such as HSP, but values <2.0 gm/dL are quite unusual in straightforward AGN and point to a process with a nephrotic syndrome component. By far, the most important (and frequently forgotten) test to obtain initially is an assessment of the complement system. This generally means obtaining a serum C3 and C4; the total hemolytic complement (“CH50”) is generally of only historical interest. Poststreptococcal AGN is characterized by a very low C3, sometimes with minimal decreases in C4 [9]. The latter is very transient and likely due to activation by Type III cryoglobulins.

The importance of a timely measurement of C3 cannot be overstressed. The hypocomplementemia of poststreptococcal AGN is evanescent, typically normalizing in six to eight weeks. On the other hand, urinary abnormalities may persist much longer. Thus, if a child with a few weeks' of abnormal urine has not had a C3 measurement earlier, it may be impossible to make a diagnosis of poststreptococcal AGN with certainty without a kidney biopsy. 

All of these tests should be easily obtained in the primary care setting and will usually identify the child for whom referral is going to be necessary.

## 5. Office Management

Some children with AGN will require immediate referral to a pediatric nephrologist. The child with severe hypertension (more than 5 mm above the 99th percentile), especially if accompanied by any neurologic complaints, must be referred immediately. Similarly, children with significant renal insufficiency should be assessed by a specialist. When AGN is accompanied by a nephrotic syndrome, the additional diagnostic and therapeutic interventions are also beyond the typical primary care practice. 

Beyond these situations, however, many such children can be reasonably managed in the primary care setting. The child with AGN in the setting of HSP, for example, who is normotensive, has normal renal function, and who is not nephrotic requires little more than careful serial observation. Although the urinary abnormalities may persist for some time after the rest of the disease has resolved, these children have little if any risk of permanent kidney injury. 

Many children with poststreptococcal AGN may also be followed in the primary care setting, but this will entail a commitment to serial examination. The major threat to such children is hypertension and its complications, and this may evolve over a few days. In otherwise typical poststreptococcal AGN with minimal hypertension (e.g., blood pressure between the 95th and 99th percentiles) and no renal failure, therapy with a loop diuretic is reasonable, with daily blood pressure rechecks. 

The urinary abnormalities in poststreptococcal AGN may persist for a long time, even a year. The best indicator of resolution of the disease is the return of the C3 level to normal. This generally occurs within 6 to 8 weeks. Persistent decrease in C3 by this time merits referral, as this could be an indicator that the “AGN” was actually the initial presentation of a more chronic process such as MPGN [10].
